# Genome-Wide Investigation of the PLD Gene Family in Tomato: Identification, Analysis, and Expression

**DOI:** 10.3390/genes15030326

**Published:** 2024-03-02

**Authors:** Xudong Guo, Wenying Zhu, Fu Wang, Hui Wang

**Affiliations:** College of Horticulture, Qingdao Agricultural University, Qingdao 266109, China; guoxudong_98@163.com (X.G.);

**Keywords:** tomato, SlPLD, expression patterns, stress

## Abstract

Phospholipase Ds (PLDs) are important phospholipid hydrolases in plants that play crucial roles in the regulation of plant growth, development, and stress tolerance. In this study, 14 PLD genes were identified in the tomato genome and were localized on eight chromosomes, and one tandem-duplicated gene pair was identified. According to a phylogenetic analysis, the genes were categorized into four subtypes: SlPLDα, β, and δ belonged to the C2-PLD subfamily, while SlPLDζ belonged to the PXPH-PLD subfamily. The gene structure and protein physicochemical properties were highly conserved within the same subtype. The promoter of all the SlPLD genes contained hormone-, light-, and stress-responsive *cis*-acting regulatory elements, but no significant correlation between the number, distribution, and type of *cis*-acting elements was observed among the members of the same subtype. Transcriptome data showed that the expression of the SlPLD genes was different in multiple tissues. A quantitative RT-PCR analysis revealed that the SlPLD genes responded positively to cold, salt, drought, and abscisic acid treatments, particularly to salt stress. Different expression patterns were observed for different genes under the same stress, and for the same gene under different stresses. The results provide important insights into the functions of SlPLD genes and lay a foundation for further studies of the response of SlPLD genes to abiotic stresses.

## 1. Introduction

Phospholipids are crucial components of cell membranes and play important roles in cellular regulation, such as signal transduction, cytoskeleton dynamics, vesicle transport, and secretion [1]. Phospholipase is a crucial enzyme in the metabolism of phospholipids. Based on the different hydrolysis sites of glycerophospholipids, phospholipases can be categorized into five types: phospholipase A1, A2, B, C, and D [2,3]. Phospholipase D (PLD) is the most important type of phospholipase in plants and specifically catalyzes the hydrolysis of phosphodiesterase bonds at the end of phospholipid molecules, producing phosphatidic acid and a free radical, such as choline and ethanolamine [4,5,6].

The PLD gene family in plants is a multigene family, with notable differences in the number of members among species [7]. In 1994, the first PLD gene was cloned in *Ricinus communis* [8]. To date, the genome-wide identification of PLD genes has been achieved in various crops, including 12 genes in *Arabidopsis thaliana*, 17 in *Oryza sativa*, 18 in *Glycine max*, 22 in maize (*Zea mays*), 32 in *Brassica napus*, 16 in *Solanum tuberosum*, 13 in *Sorghum bicolor*, and 59 in *Medicago sativa* [9,10,11,12,13,14,15,16]. In the dicotyledon *A. thaliana*, the 12 PLD genes are classified into six subtypes, namely, α, β, γ, δ, ζ, and ε [17]. In the monocotyledon *O. sativa*, the 17 PLD genes are classified into the subtypes α, β, δ, κ, ζ, and φ [10]. Plant PLD proteins contain two structural domains: a C-terminal catalytic domain containing two HKD motifs, and an N-terminal PX/PH or C2 domain [18,19]. According to their N-terminal domains, PLDs are classified into two subfamilies: PX/PH-PLD and C2-PLD [20]. The PLDα, β, γ, δ, and ε subtypes of Arabidopsis belong to the C2-PLD subfamily, whereas PLDζ is classified as being in the PX/PH-PLD subfamily [17,21].

The different subtypes of PLD have different functions. Arabidopsis *PLDα1* participates in the regulation of guard cells [22]. The overexpression of *AnPLDα* cloned from *Ammopiptanthus nanus* in Arabidopsis *PLDα1*-deficient mutants can increase the salt tolerance of the mutants [23]. The expression level of *TaPLDα* in wheat increases in several abiotic stresses. The overexpression of *TaPLDα* in Arabidopsis results in significantly improved drought tolerance [24]. The heterologous expression of *GhPLDδ* cloned from *Gossypium hirsutum* can enhance salt tolerance in Arabidopsis; *GhPLDδ* participates in regulating *G. hirsutum* defense against *Verticillium dahliae* infection [25]. An increase in the expression of *AtPLDα* in Arabidopsis enhances stomatal closure [26]. In potato (*S. tuberosum*), *StPLDα5* is specifically expressed in the stamens, and *StPLDα5* is associated with tolerance to salt, drought, and heat [14]. The expression of *OsPLDβ1* promotes ABA signaling by activating SAPK, thereby negatively affecting seed germination [10]. In maize, the expression of *ZmPLDα3* in the root system responds to drought stress. Moreover, *ZmPLDα3* plays an important role in the haploid breeding of maize. The loss of function of *ZmPLDα3* can trigger haploid induction in maize maternal plants [12,27].

Tomato (*Solanum lycopersicum*) fruit are of a high nutritional value and have a unique flavor. Tomato is among the most important vegetable crops worldwide and is a model crop for molecular biology research [28,29,30,31]. Although preliminary exploration of the tomato PLD gene family was conducted as early as 2001, with a total of five PLD genes (*LePLDβ2*, *LePLDβ1*, *LePLDα3*, *LePLDα2*, *LePLDα1*) identified and cloned [32], there is a lack of comprehensive surveys into the tomato PLD gene family. Therefore, in this study, we identified SlPLD genes in the latest tomato genome and systematically analyzed the physicochemical properties, phylogenetic relationships, gene structure, structural domains, promoter *cis*-acting elements, and organ expression patterns. In addition, we investigated the transcript abundance by qRT-PCR analysis under exposure to four abiotic stresses. This study lays a foundation for further investigation of the functions of SlPLD gene family members.

## 2. Materials and Methods

### 2.1. Plant Materials and Treatment Methods

This study used *Solanum lycopersicum* ‘Ailsa Craig’ as the experimental material. Tomato seedlings were grown in an artificial climate chamber with a photoperiod of 16 h/8 h (light/dark) and temperature of 25 ± 1 °C. To investigate the expression patterns of SlPLD genes under four abiotic stresses (cold, salt, ABA, and drought), we subjected tomato seedlings with relatively uniform and healthy growth at the four-leaf and one-heart stages to stress treatment, and collected leaf samples at 0, 3, 6, 9, 12, and 24 h after stress initiation. For cold treatment, seedlings were transferred to a 4 °C (16 h light/8 h dark) incubator. For salt treatment, the roots of seedlings were submerged in 200 mM NaCl solution. For ABA treatment, seedlings were sprayed with 100 μM ABA solution. To simulate drought stress, the roots of seedlings were soaked with 20% polyethylene glycol 6000 solution. Three biological replicates were established for each treatment and each replicate consisted of 12 seedlings. Leaves were collected from the same part of the seedlings and immediately frozen in liquid nitrogen.

### 2.2. Identification of the PLD Gene Family in Tomato

The tomato ITAG 4.0 protein file, gff3 file, and SL4.0 genome file were acquired from the following website (https://solgenomics.net/projects/tomatodisease/; accessed on 7 June 2023) [33]. The 12 AtPLD protein sequences were downloaded from the TAIR database (https://www.arabidopsis.org/index.jsp; accessed on 7 June 2023). The Hidden Markov Model (HMM) configuration file for the PLD domain (PF00614) was downloaded from the Pfam database (https://pfam.xfam.org/; accessed on 8 June 2023) [34]. Next, the SlPLD candidate genes were searched in the tomato protein sequence file using the Simple HMM search function of TBtools (v2.012) [35]. Then, using the AtPLD protein sequences as query sequences, the BlastP algorithm was used to search the tomato protein sequence file. The candidate SlPLD members selected by these two methods were submitted to SMART (http://smart.embl-heidelberg.de/; accessed on 8 June 2023). Members containing the PH/PX or C2 domain and two HKD domains were selected for further analysis. The physicochemical properties of all selected SlPLD protein sequences were predicted and analyzed using the Expasy database (https://web.expasy.org/protparam/; accessed on 9 June 2023).

### 2.3. Phylogenetic Relationships, Gene Structure, Domains, Protein Motifs, and Cis-Regulatory Elements of the SlPLD Gene Family

The potato protein sequence file, potato genome file, and potato gff3 file were downloaded from the Spud DB database (http://solanaceae.plantbiology.msu.edu; accessed on 11 June 2023). The Arabidopsis protein sequence file, Arabidopsis genome file, and Arabidopsis gff3 file were downloaded from the TAIR database. In MEGA11, the amino acid sequences from tomato, Arabidopsis, and potato were used to generate a multiple sequence alignment using Muscle, and a phylogenetic tree was constructed using the maximum likelihood method with 1000 bootstrap replicates.

The intron and exon positions were determined based on information in the tomato ITAG 4.0 gff3 file. Conserved motifs in the SlPLD protein sequences were identified using the MEME Suite (https://meme-suite.org/; accessed on 9 June 2023); the maximum number of motifs was set as 10 and the other parameters were set as the default values [36]. The Pfam v34.0 database and the CD-search function on the NCBI website (https://www.ncbi.nlm.nih.gov/; accessed on 15 June 2023) were used to analyze the structural domains of the protein sequences. The 2000 bp region upstream of the start codon was extracted as the promoter sequence of each SlPLD gene. Prediction of *cis*-acting elements in the promoter regions was performed using the Search for CARE tool in the PlantCARE database (https://bioinformatics.psb.ugent.be/webtools/plantcare/html/; accessed on 20 June 2023) [37]. All analytical results were visualized using TBtools [35].

### 2.4. Synteny Analysis of SlPLD Genes

The tomato genome and gff3 files were imported into the MCScan software (Python version) to analyze gene duplication events. The One-step MCScanX function of TBtools was used to explore the microsynteny among SlPLD, AtPLD, and StPLD genes.

### 2.5. Expression Patterns of SlPLD Genes

We obtained transcriptome data for tomato ‘Heinz’ from the publicly accessible Tomato Functional Genomics database (http://ted.bti.cornell.edu; accessed on 5 July 2023) and the Tomato eFP Browser (http://bar.utoronto.ca/efp_tomato/cgi-bin/efpWeb.cgi; accessed on 6 September 2023), which comprised data for the whole roots, leaves, unopened flower buds, fully opened flowers, 1 cm fruit, 2 cm fruit, 3 cm fruit, mature green fruit, breaker fruit, and breaker + 10 fruit [38].

### 2.6. RNA Extraction and Quantitative Real-Time PCR Analyses

Total RNA from tomato leaves was extracted using the Plant RNA Kit (Yeasen, Shanghai, China). First-strand cDNA synthesis was performed with the AdvanceFast One-step RT-gDNA Digestion SuperMix for RT-PCR (Yeasen) in accordance with the manufacturer’s instructions. qRT-PCR was conducted with the SYBR Green Master Mix (11184ES03, Yeasen) using a LightCycler 480 instrument (Roche, Basel, Switzerland). Specific primers for SlPLD genes were designed with NCBI Primer-BLAST (https://www.ncbi.nlm.nih.gov/tools/primer-blast/; accessed on 10 September 2023) and are listed in Supplementary Table S1. The tomato β-Actin gene (Solyc03g078400) was used as an internal control [39]. Three biological replicates were analyzed for each group and three technical replicates were analyzed for each reaction. The relative expression level of the target genes was calculated using the 2^−ΔΔ*C*t^ method.

## 3. Results

### 3.1. Identification and Analysis of SlPLD Family Members in Tomato

To identify SlPLD genes in the tomato genome, a BlastP search was performed using the Arabidopsis PLD amino acid sequences as queries and then screened with the Simple HMM search function of TBtools based on the PLD gene domain (PF00614). SMART was used to remove the sequences that lacked an HKD domain or included only one HKD domain. A total of 14 PLD genes were found in the tomato genome and were distributed on eight chromosomes. The SlPLD genes were named according to the subtype classification and gene location. The physicochemical properties of the genes are listed in Table 1. The SlPLD genes encoded proteins ranging from 807 to 1106 amino acids, the protein molecular weight ranged from 92,027.6 to 125,961.43 Da, and the isoelectric point ranged from 5.39 to 8.44.

### 3.2. Phylogenetic Analysis of SlPLD Proteins

**Figure 1:**
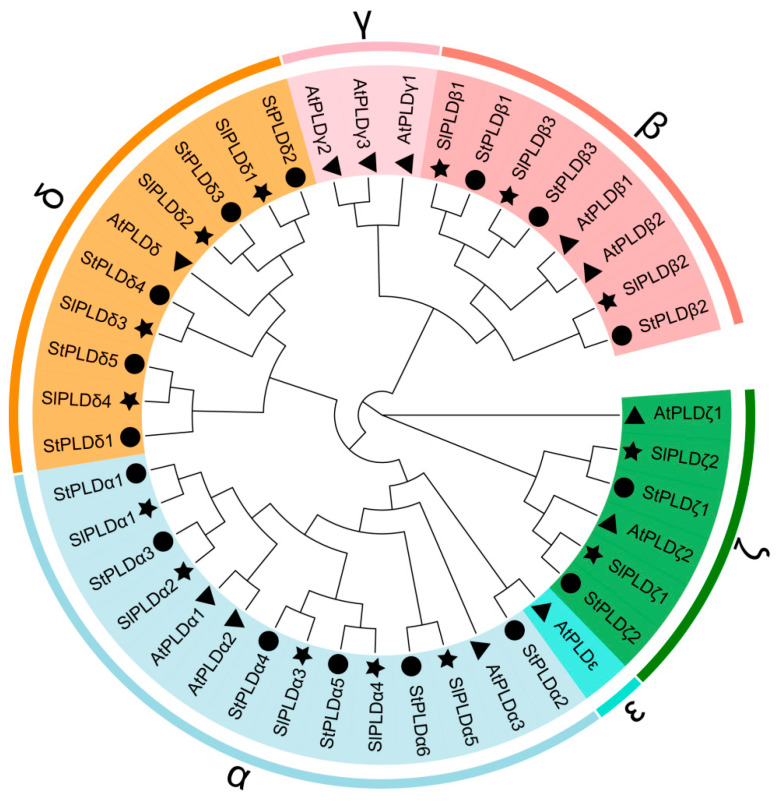
Phylogenetic tree of Phospholipase D (PLD) proteins from tomato, Arabidopsis, and potato. The shading color indicates different PLD subtypes. The symbols ●, ▲, and ★ indicate PLDs of potato, Arabidopsis, and tomato, respectively.

To explore the phylogenetic relationship of SlPLD family members, a phylogenetic tree was constructed based on a multiple sequence alignment of amino acid sequence data for 42 PLD proteins, consisting of 12 from Arabidopsis, 16 from potato, and 14 from tomato (Figure 1). The 42 proteins were resolved into six subtypes, namely, α, β, γ, δ, ε, and ζ. Tomato and potato, both members of Solanaceae, lacked the γ and ε subtypes. The SlPLD genes comprised five α-subtype members, three β-subtype members, four δ-subtype members, and two ζ-subtype members. The PLDζ subtype for each of the three species included in the analysis comprised only two members.

### 3.3. Gene and Domain Structure Analysis

**Figure 2:**
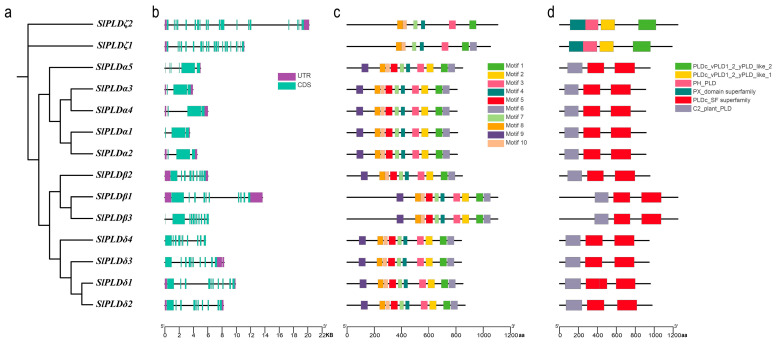
Sequence structure analysis of SlPLD proteins in tomato. (**a**) Phylogenetic tree of the 14 SlPLD proteins. (**b**) Motif distribution of the SlPLD proteins. Different motifs (1–10) are indicated by different colors. (**c**) Exon–intron structure of the SlPLD proteins. The “aa” in the figure is short for amino acid. (**d**) Domain distribution of the SlPLD proteins. Different domains are indicated by different colors. The “aa” in the figure is short for amino acid.

To examine the diversity of gene structures among members of the tomato PLD gene family and among subtypes, we constructed a phylogenetic tree using the amino acid sequences of the 14 SlPLD proteins (Figure 2a). The characteristics of the exons and introns in the SlPLD genes differed among the subtypes, whereas the domain structure within the same subtype was relatively conserved (Figure 2b). The number of exons in the SlPLDζ members was 20 and 21. The SlPLDα members predominantly had three exons, with only *SlPLDα4* containing six exons. Except for *SlPLDβ3*, which included 11 exons, all other members of the SlPLDβ and SlPLDδ subtypes contained 10 exons. In addition, most members of the same subtype had similar numbers of exons.

A total of 10 different motifs were identified (Figure 2c). According to their phylogenetic relationships, proteins of the same subtype had similar motifs and arrangements, whereas notable differences in motifs were detected between different subtypes. All members of the SlPLDα, β, and δ subtypes contained motifs 1–10 with the exception of *SlPLDζ1*, which lacked motifs 2, 5, and 9, and *SlPLDζ2*, which lacked motifs 2, 5, 6, and 9. The SlPLDζ subtype included only motifs 1, 3, 4, 7, 8, and 10.

We retrieved the conserved domains contained in the SlPLD family members from the Pfam database. The 14 SlPLD proteins contained a total of six conserved domains associated with phospholipase D (Figure 2d). Based on their phylogenetic relationships, members of each subfamily shared the same domain. The SlPLD family members are divided into two subfamilies based on their different N-terminal domains. The SlPLDα, β, and δ subtypes contained the C2 domain characteristic of the C2-PLD subfamily, whereas SlPLDζ members contained the PH and PX domains that typify the PHPX-PLD subfamily.

### 3.4. Chromosomal Distribution and Collinearity Analysis of SlPLD Genes

**Figure 3:**
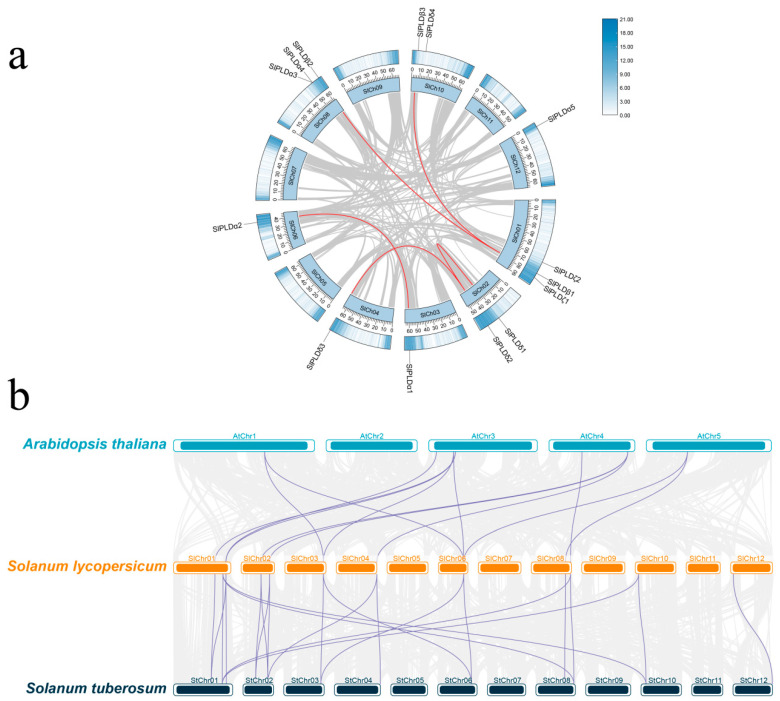
Gene duplication analysis of tomato SlPLD family members. (**a**) Intraspecific collinearity analysis of tomato. The innermost circle represents the 12 chromosomes of tomato; the graduated scale beside each chromosome indicates the length of the chromosome. The outermost circle represents a heatmap of the gene density on each chromosome. Gray lines indicate all syntenic blocks in the tomato genome; the duplicated SlPLD gene pairs are connected by red lines. (**b**) Syntenic relationships of the *PLDs* among Arabidopsis, potato, and tomato. Gray lines indicate all syntenic blocks present in their respective genomes. Purple lines indicate the homology and evolutionary links of *PLDs*.

Chromosomal localization analysis revealed that the 14 SlPLD genes were irregularly distributed on eight chromosomes (Figure 3a). The highest number of SlPLD genes were localized on chr1 and chr8, with three each, followed by chr2 and chr10, each of which had two SlPLD genes. A single SlPLD gene was localized on chr3, 4, 6, and 12. The gene density near the end of the chromosome arms was higher, whereas the gene density in the centromeric regions was lower.

To identify gene duplication events in the SlPLD gene family, a collinearity analysis was conducted on the tomato genome using TBtools. In the SlPLD gene family, five syntenic gene pairs were identified, and one tandem-duplicated gene pair was detected on chromosome 8 comprising *SlPLDα3* and *SlPLDα4* (Figure 3a).

The collinearity analysis was performed on the PLD families of tomato and Arabidopsis, and of tomato and potato, respectively (Figure 3b). Between tomato and Arabidopsis, excluding *SlPLDβ1*, *SlPLDβ3*, *SlPLDα4*, *SlPLDα5*, and *SlPLDδ4*, all other SlPLD genes were identified as homologous to genes in Arabidopsis, with a total of 13 homologous gene pairs identified (Table S2). The five aforementioned genes may be unique to tomato evolution. Among the 13 gene pairs, only the pair of *SlPLDβ2* and *AtPLDγ2* corresponded one-to-one, indicating that this gene pair may have a common origin before species divergence. Between tomato and potato, only *SlPLDδ4* lacked homologous genes in the potato genome and a total of 22 homologous gene pairs were identified (Table S3). This result implied that *SlPLDδ4* may have a unique function in tomato.

### 3.5. Cis-Elements in the Promoter of SlPLD Genes

**Figure 4:**
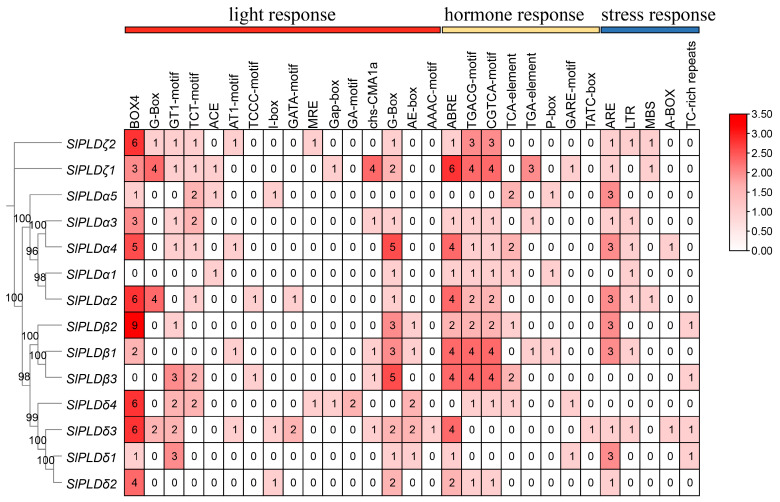
Predicted *cis*-acting elements in the promoter region of tomato SlPLD genes. The number in each box is the number of *cis*-acting elements.

Based on the functions of different *cis*-acting elements, we screened 29 major putative *cis*-elements and grouped them into three categories: 16 that respond to light, five that respond to stress, and eight hormone response elements (Figure 4). The gene *SlPLDζ1* had the most *cis*-acting elements (37), whereas *SlPLDα1* contained the fewest *cis*-acting elements (8). Considering all 14 SlPLD genes, the most abundant light-responsive element was BOX4 (52); among the hormone-responsive elements, 24 TGACG-motifs and 24 CGTCA-motifs that respond to methyl jasmonate, and 34 ABA-responsive elements, were detected; and among the stress-responsive elements, elements responsive to anaerobic induction were the most frequent (24). However, no significant correlation between the number, distribution, and type of *cis*-acting elements was observed among different members of the same subtype. Thus, we speculate that SlPLD genes may participate in multiple abiotic stress and hormone regulatory processes in tomato.

### 3.6. Expression Patterns of SlPLD Genes Revealed by Transcriptome Analysis

**Figure 5:**
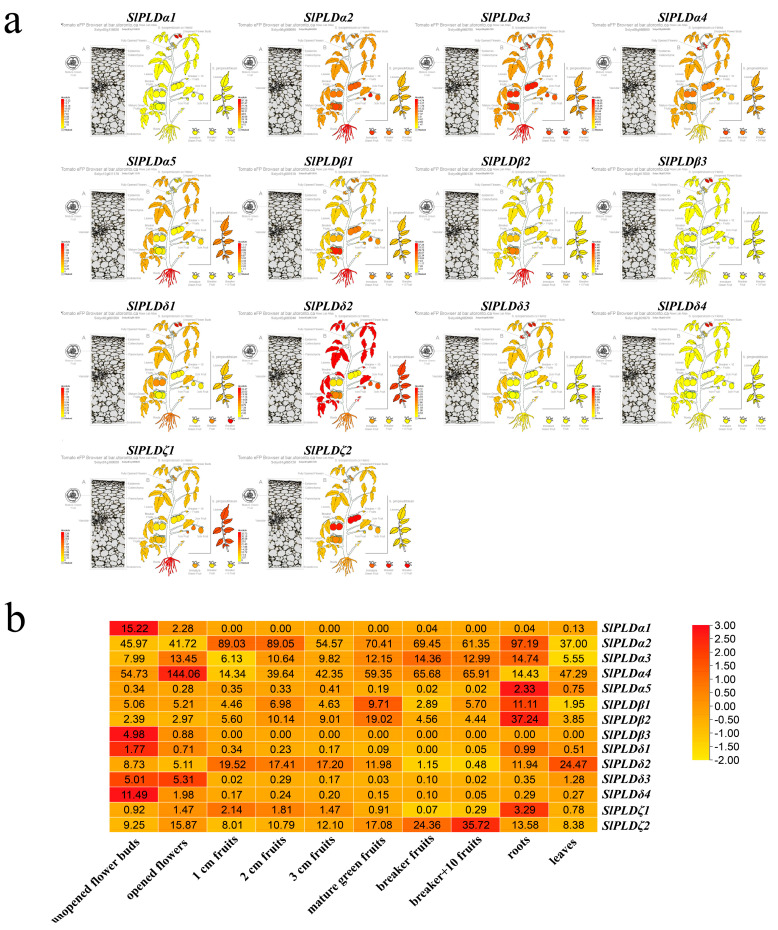
The expression patterns of SlPLD genes. (**a**) Expression patterns of tomato SlPLD genes at different developmental stages and in different tissues analyzed with the Tomato eFP Browser. The part A is the expression pattern of SlPLD genes in different tissues of pericarp. In part B, the left side is the expression pattern of SlPLD genes in various organs of *S. lycopersicum* cv Heinz, and the right side is the expression pattern of SlPLD genes in *S. pimpinellifolium* leave and fruit. (**b**) The heatmap represents SlPLD gene expression in tomato ‘Heinz’ at different developmental stages and in different tissues. The number in each box is the FPKM value.

To evaluate the tissue specificity of the SlPLD genes, we used the Tomato eFP Browser and publicly accessible RNA-sequencing data to compare the expression levels of the SlPLD genes in different tissues (Figure 5a). The genes *SlPLDα1*, *SlPLDβ3*, and *SlPLDδ1* were not detected in all tissues. *SlPLDδ1* was detected in all tissues except fruit at the breaker stage, whereas *SlPLDα1* was detected only in unopened flower buds, opened flowers, roots, and leaves, but the expression level was low in roots and leaves. *SlPLDβ3* was expressed only in unopened flower buds and opened flowers. Thus, *SlPLDα1* and *SlPLDβ3* showed flower-specific expression. The other 11 *SlPLD* genes were expressed in various tissues. At the individual gene level, *SlPLDα5*, *SlPLDβ1*, *SlPLDβ2*, and *SlPLDζ1* were predominantly expressed in roots; *SlPLDα1*, *SlPLDβ3*, *SlPLDδ1*, and *SlPLDδ4* were mainly expressed in unopened flower buds; *SlPLDδ3* and *SlPLDα4* were mostly expressed in opened flowers; *SlPLDδ2* was mainly expressed in leaves; and *SlPLDζ2* was predominantly expressed in breaker + 10 fruit (Figure 5b). These expression patterns may be associated with the role of the genes in different tissues.

### 3.7. Expression Profiles of SlPLD Genes in Response to Abiotic Stress Exposure

**Figure 6:**
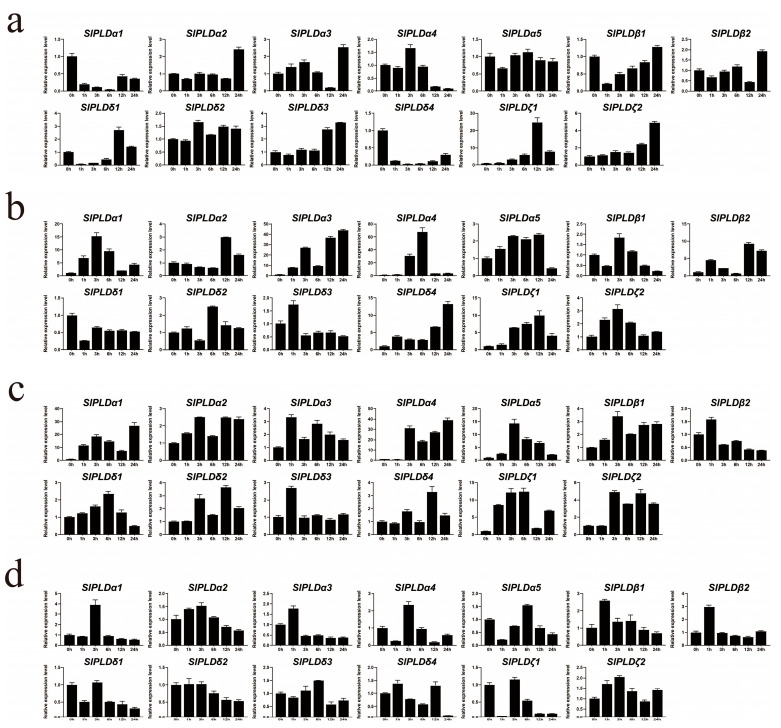
Expression patterns of tomato SlPLD genes in the leaf under exposure to abiotic stress. (**a**) Cold (4 °C), (**b**) salt (200 mM NaCl), (**c**) drought (20% PEG 6000), and (**d**) abscisic acid (100 μM).

To investigate the changes in the expression of the SlPLD genes in response to abiotic stress, the relative expression levels were verified at 1, 3, 6, 9, 12, and 24 h of exposure to cold, salt, drought, and ABA, using expression at 0 h as the control. *SlPLDβ3* was not detected in response to the four stress treatments, consistent with the aforementioned transcriptome data. The qPCR results indicated that all other genes responded to the treatments and that the SlPLD genes responded differentially to the different abiotic stresses. Under cold stress (Figure 6a), *SlPLDα1* and *SlPLDδ4* exhibited a continuous downregulation trend from 0 to 6 h, followed by a slight upregulation trend from 6 to 24 h. *SlPLDδ3* and *SlPLDζ2* showed a continuous upregulation trend. The expression patterns of *SlPLDα5* and *SlPLDδ2* were similar, and almost did not change during low-temperature stress. For *SlPLDα2*, *SlPLDα3*, *SlPLDβ1*, *SlPLDβ2*, *SlPLDδ3*, and *SlPLDζ2*, the highest expression level was observed at 24 h compared with that of the control. *SlPLDζ1* and *SlPLDζ2* exhibited the strongest response to cold stress.

Under salt stress (Figure 6b), the expression levels of most genes, especially *SlPLDα1*, *SlPLDα4*, *SlPLDα5*, *SlPLDδ3*, *SlPLDζ1*, and *SlPLDζ2*, initially increased but thereafter decreased, whereas *SlPLDδ4* showed a continuous upregulation trend. The response of *SlPLDα1*, *SlPLDα3*, *SlPLDα4*, *SlPLDβ2*, *SlPLDδ4*, and *SlPLDζ1* was strong, with the highest gene expression level being 10, 43, 67, 9, 13, and 9 times higher than that of the control, respectively.

Under drought stress (Figure 6c), all the SlPLD genes were upregulated. Among them, *SlPLDα1*, *SlPLDα4*, *SlPLDα5*, *SlPLDζ1*, and *SlPLDζ2* were significantly upregulated. The expression level of *SlPLDα1, SlPLDα3, SlPLDδ3,* and *SlPLDζ1* increased rapidly after one hour of drought stress. The relative expression levels of all 13 SlPLD genes showed a roughly similar trend of change. 

Under ABA stress (Figure 6d), excluding *SlPLDβ2* and *SlPLDζ2*, the expression levels of the other 12 genes were lower than those of the control at 24 h. *SlPLDα1*, *SlPLDα3*, *SlPLDβ1*, and *SlPLDβ2* showed a similar expression pattern (an abrupt increase at 1 or 2 h, followed by markedly lower expression levels). The expression levels of *SlPLDα2* and *SlPLDδ2* showed a similar pattern (initially high or a gradual increase, peaking at 2 h, followed by a gradual decline thereafter).

## 4. Discussion

Phospholipase Ds are enzymes crucial to membrane dynamics that hydrolyze membrane phospholipids to generate phosphatidic acid and participate in many developmental processes, such as plant growth and defense reactions [17,40,41,42]. In the present study, a genome-wide analysis identified 14 members of the PLD genes in tomato and their expression patterns under exposure to four abiotic stresses were analyzed. The number of SlPLD genes in tomato is similar to the number identified in each of *A. thaliana*, *O. sativa*, *G. max*, *Z. mays*, *S. tuberosum*, and *S. bicolor*, but differs markedly from the number in *B. napus* and *M. sativa* [9,10,11,12,13,14,15,16]. We infer that the numerical difference reflects that *B. napus* and *M. sativa* are tetraploid [16,43], while the other species mentioned are diploid. The analysis of gene duplication events revealed the presence of only one tandem-duplicated gene pair in the tomato genome, duplication events involving PLD genes have not been reported in Arabidopsis, and only two pairs of tandem-duplicated PLD genes were detected in potato. Therefore, gene duplication events have directly led to the lower diversity of PLD genes in tomato compared with tetraploid plants (Figure 3a). 

The 14 SlPLD genes were classified into four subtypes, namely, SlPLDα, β, δ, and ζ, based on a phylogenetic analysis including PLD genes from *A. thaliana* and *S. tuberosum*. The subtypes γ and ε present in *A. thaliana* were not represented in the tomato genome (Figure 1). Based on the topology of the evolutionary tree, the SlPLD and StPLD genes showed the highest homology. The analysis of the physicochemical properties of the SlPLD genes revealed that the protein lengths of the SlPLDα, β, δ, and ζ subtypes were 807–848 bp, 846 or 1106 bp, 839–866 bp, and 1052 or 1106 bp, respectively (Table 1). The protein sequence length, gene structure, and distribution of the conserved motifs were similar among members of the same subtype. Based on the N-terminal structural domain, the tomato PLD genes were classified into two subfamilies, C2-PLD and PX/PH-PLD (Figure 2d). A novel subfamily, SP-PLD, has been recorded in the monocotyledonous plant *O. sativa* [10]. The SlPLDα, β, and δ subtypes were members of the C2-PLD subfamily, whereas the subtype SlPLDζ belongs to the PX/PH-PLD subfamily (Figure 2d). All of these findings suggest that SlPLD genes are evolutionarily conserved and may have similar functions to those reported for *PLD* genes in other plant species. The PLD gene family of potato, which belongs to the Solanaceae family, the same as tomato, is also divided into four subtypes (α, β, δ, and ζ). The PLD gene family gene structures of tomato and potato are similar; the gene length of subtype α is short with fewer exons, while the gene length of subtype δ is short with more exons. The subtype β consists of three members, while the subtype ζ has two members and the gene lengths are longer than other subtypes [14].

The tissue-specific expression analysis revealed that the 14 SlPLD genes were expressed in various tissues, but marked differences in expression levels were observed between different genes in the same tissue, and in different tissues for the same gene (Figure 5). Five PLD genes were identified and cloned in previous research results; each PLD in tomato has different expression patterns in different organs [32]. *SlPLDα5*, *SlPLDβ1*, *SlPLDβ2*, and *SlPLDζ1* were mainly expressed in the roots. The overexpression of *AtPLDζ1* participates in the elongation of root hairs [44]. *AtPLDζ1* and *AtPLDζ2* play roles in root growth and development under nutrient deficiency [45]. This finding implies that *SlPLDζ1* may perform functions mainly in the roots. *SlPLDα1*, *SlPLDα4*, *SlPLDβ3*, *SlPLDδ1*, *SlPLDδ3,* and *SlPLDδ4* were most highly expressed in the flower, suggesting that they play roles in tomato reproductive growth. The loss of function of *ZmPLDα3*, which is specifically expressed in the pollen, causes haploid induction in maize. We identified a pollen-specific subtype α gene, *SlPLDα1*, in tomato. Therefore, we speculate that the loss of function of *SlPLDα1* may lead to haploid induction in tomato [12,27]. However, this hypothesis will require verification in a future study. The expression pattern of the PLD gene family in potato belonging to the Solanaceae family is different from that in tomato [14], which may be due to differences between species and living environments.

We analyzed the expression profiles of the SlPLD genes under cold, salt, drought, and ABA stresses using qRT-PCR. The experimental results indicated that the SlPLD genes actively responded to the four abiotic stresses, particularly to salt stress; different expression patterns were observed for different genes under the same stress, and for the same gene under different stress treatments (Figure 6). Under salt stress, most of the SlPLD genes exhibited significant responses (Figure 6b). In Arabidopsis, *AtPLDα1* is activated under salt and drought stress [46]. *SlPLDα1* and *SlPLDα2* of tomato are orthologs of *AtPLDα1*, and exhibited strong responses to salt and drought stress, of which *SlPLDα1* showed a significantly stronger response than *SlPLDα2* (Figure 6a,b). The reason for this phenomenon may be that *SlPLDα1* and *SlPLDα2* evolved and were expressed in different tissues, resulting in the functional division of labor (Figure 5). Similar results have been reported for *Cicer arietinum*, where *CaPLDα1* is strongly expressed in response to drought stress [47]. *SlPLDζ1* was significantly upregulated under cold stress and may play a role in tolerance to low temperature (Figure 6a). The *SlPLDβ1* and *SlPLDβ2* genes can quickly respond to ABA stress, and after 1 h of ABA stress, the expression levels of *SlPLDβ1* and *SlPLDβ2* increase by 2 to 3 times (Figure 6d). The accumulation of *LePLDβ1* mRNA in tomato leaves and tomato cell suspensions peaked at 1 to 2 h after xylanase stress [32]; by aligning protein sequences, *LePLDβ1* represents *SlPLDβ2* in this study (Table S5). Silencing *LePLDβ1* can increase the activity of polyphenol oxidase, reduce the secretion of LeXYL2 and promote the synthesis of XEGIP in tomato cell suspensions; *LePLDβ1*-silenced cells enhanced reactive oxygen species response under xylanase stress [48]. The SlPLD genes identified have the potential to enhance the tolerance of tomato to the four tested stresses, and are candidate genes for the improvement of the stress tolerance of other crops.

## 5. Conclusions

We conducted a genome-wide identification and analysis of tomato phospholipase D family members. Fourteen SlPLD genes were identified in the tomato genome. Based on their phylogenetic relationships, the SlPLD genes were classified into four subtypes: α, β, δ, and ζ. According to their structural domains, the SlPLD genes belonged to two subfamilies: C2-PLD and PX/PH-PLD. The 14 SlPLD genes were distributed on eight chromosomes. Members of the same subtype shared similar physicochemical properties, gene structures, conserved motifs, and domains. One tandem-duplicated gene pair was detected in the SlPLD gene family. The SlPLD genes contained multiple *cis*-acting elements that respond to light, hormones, and abiotic stress. A qRT-PCR analysis showed that most of the SlPLD genes responded positively to cold, salt, drought, and ABA stress. The results of this study lay a foundation for future functional investigations of the SlPLD gene family and their utilization in molecular breeding in tomato. In order to gain a deeper understanding of the changes in the PLD gene family in tomato evolution, further analysis of PLD gene families is needed for *Solanum pennellii*, *Solanum lycopersicoides,* and *Solanum pimpinellifolium.*

## Figures and Tables

**Table 1:** Basic information for the 14 SlPLD genes identified in this study.

Gene Name	Gene ID	Gene Locus	Protein Length	Molecular Weight	PI
*SlPLDα1*	Solyc03g116620.3.1	Chr03:60414826..60418303+	811	92,638.49	5.41
*SlPLDα2*	Solyc06g068090.3.1	Chr06:39833679..39838185+	809	92,200.33	5.39
*SlPLDα3*	Solyc08g066790.4.1	Chr08:53735254..53739172+	807	92,750.81	6.22
*SlPLDα4*	Solyc08g066800.4.1	Chr08:53752165..53758183+	807	92,027.6	5.63
*SlPLDα5*	Solyc12g011170.3.1	Chr12:4055864..4060853+	848	96,687.18	6.58
*SlPLDβ1*	Solyc01g091910.4.1	Chr01:77680828..77694493+	1106	123,113.22	6.54
*SlPLDβ2*	Solyc08g080130.3.1	Chr08:61577138..61583167+	846	95,641.76	6.75
*SlPLDβ3*	Solyc10g017650.3.1	Chr10:5319066..5325156+	1106	124,037.59	7.24
*SlPLDδ1*	Solyc02g061850.4.1	Chr02:31366227..31376103-	850	97,673.09	6.67
*SlPLDδ2*	Solyc02g083340.4.1	Chr02:44778224..44786418-	866	98,649.5	6.87
*SlPLDδ3*	Solyc04g082000.4.1	Chr04:63789988..63798274+	839	95,664.13	7.09
*SlPLDδ4*	Solyc10g024370.3.1	Chr10:13291114..13296807+	839	94,118.42	8.44
*SlPLDζ1*	Solyc01g100020.4.1	Chr01:82361097..82372211+	1052	120,394.27	6.08
*SlPLDζ2*	Solyc01g065720.4.1	Chr01:65078959..65099137-	1106	125,961.43	6.44

## Data Availability

Data are contained within the article and Supplementary Materials.

## Supplementary Materials

The following supporting information can be downloaded at https://www.mdpi.com/article/10.3390/genes15030326/s1, Table S1: Sequences of primers used in qRT-PCR analysis; Table S2: Syntenic gene pairs between *Solanum lycopersicum* and *Arabidopsis thaliana*; Table S3: Syntenic gene pairs between *Solanum lycopersicum* and *Solanum tuberosum*; Table S4: List of tandem duplication gene pairs of the SlPLD gene family; Table S5: Protein sequence list of 14 SlPLD genes; Table S6: List of 10 motifs with basic information; Figure S1: Alignment of SlPLD family protein sequences; Figure S2: Logos of 10 motifs in SlPLD.
