# Supplementary figures and images for: Genome-Wide Investigation of the PLD Gene Family in Tomato: Identification, Analysis, and Expression
Source: Genes (Basel). 2024 Mar 2;15(3):326. doi: 10.3390/genes15030326 (PMC10970076; doi:10.3390/genes15030326)

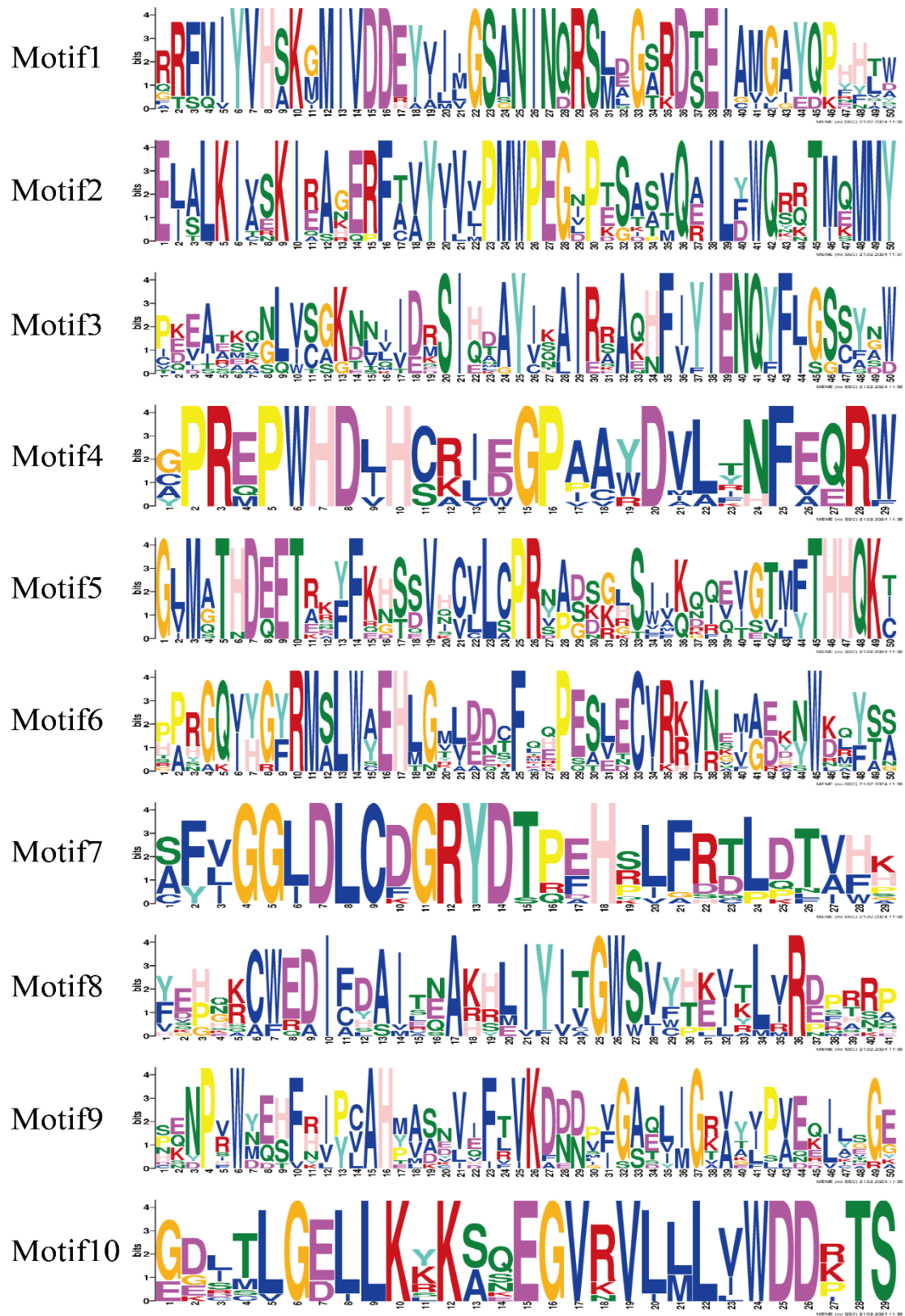

**Figure S2.** Logos of 10 motifs in tomato.

Supplement: Supplementary file 1 [file genes-15-00326-s001.zip › Supplementary Materials-0301/Figure S2.pdf]
