# Supplementary material for: Genome-Wide Investigation of the PLD Gene Family in Tomato: Identification, Analysis, and Expression
Source: Genes (Basel). 2024 Mar 2;15(3):326. doi: 10.3390/genes15030326 (PMC10970076; doi:10.3390/genes15030326)
Supplement: Supplementary file 1 [file Figure S1.pdf › Supplementary Materials-0301/Figure S1.pdf]

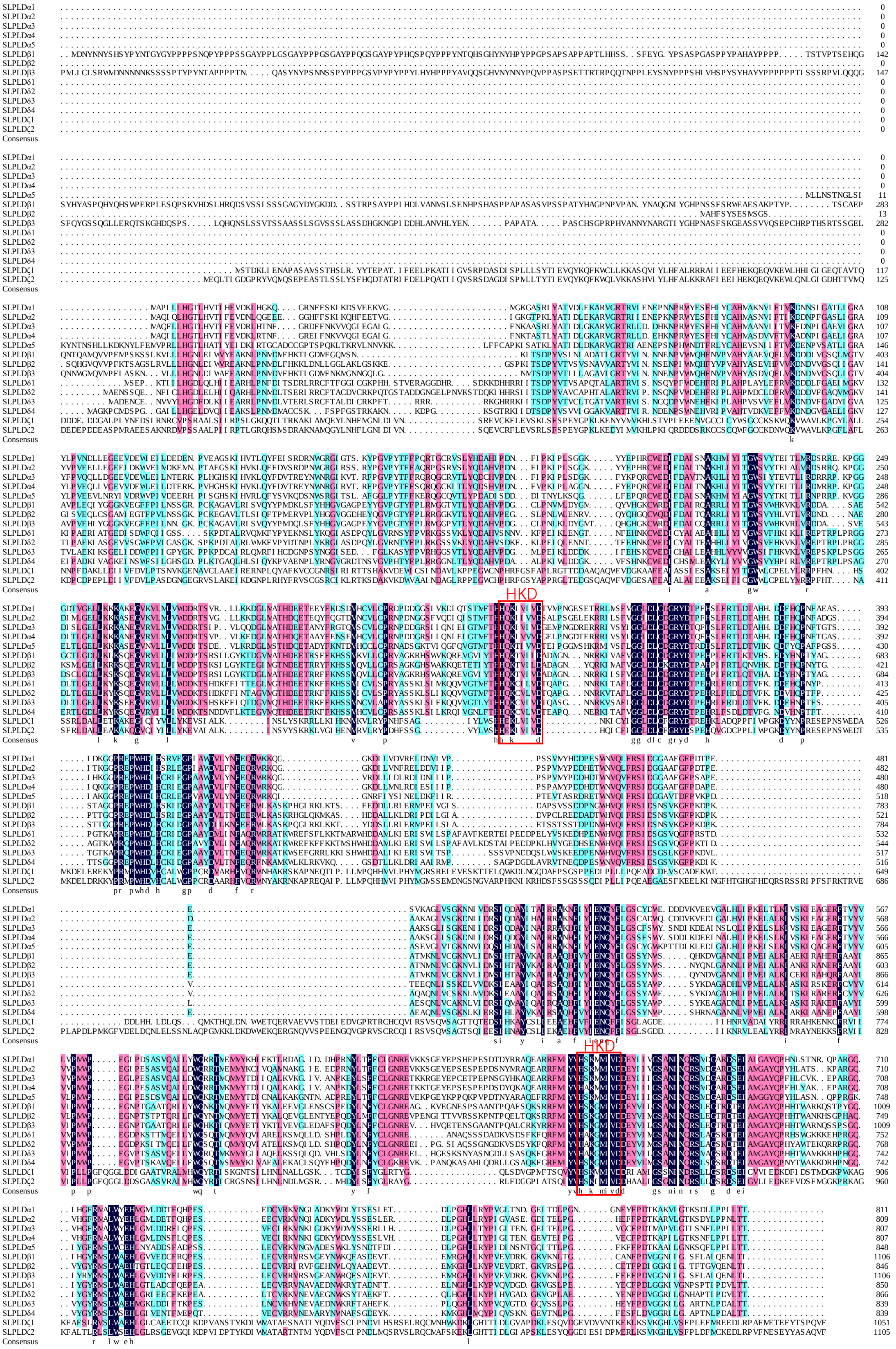

Figure S1. Alignment of SlPLD family protein sequences. Amino acids marked in dark blue indicate 100% sequence identity, pink indicates ≥75% identity, and light blue indicates ≥50% identity. The red boxes indicate the HKD domain of the PLD gene family.
